# Global Leadership Initiative on Malnutrition Criteria and Immunonutritional Status Predict Chemoadherence and Survival in Stage II/III Gastric Cancer Treated with XELOX Chemotherapy

**DOI:** 10.3390/nu16203468

**Published:** 2024-10-14

**Authors:** Jong Hyuk Yun, Geum Jong Song, Myoung Won Son, Moon Soo Lee

**Affiliations:** Department of Surgery, Soonchunhyang University Cheonan Hospital, Soonchunhyang University College of Medicine, 31 Suncheonhyang 6-gil, Cheonan 31151, Republic of Korea; 109206@schmc.ac.kr (J.H.Y.); gjsong@schmc.ac.kr (G.J.S.); mwson@schmc.ac.kr (M.W.S.)

**Keywords:** gastric cancer, GLIM criteria, chemotherapy adherence, relative dose intensity (RDI), survival, malnutrition

## Abstract

Backgroud: Adjuvant chemotherapy is crucial for the treatment of advanced gastric cancer. However, various factors negatively impact chemoadherence, with malnutrition after gastrectomy being a critical determinant. This study aims to analyze the impact of malnutrition, assessed through the Global Leadership Initiative on Malnutrition (GLIM) and other immunonutritional indices, on chemoadherence and its subsequent effect on survival. Methods: This retrospective study included 116 patients who underwent curative gastrectomy and received oxaliplatin and capecitabine (XELOX). Preoperative nutritional status was assessed using the GLIM criteria along with other immunonutritional indices, such as the prognostic nutritional index (PNI), C-reactive protein-to-albumin ratio (CAR), neutrophil–lymphocyte ratio (NLR), controlling nutritional status (CONUT) score, and modified Glasgow Prognostic Score (mGPS). Chemotherapy adherence was measured using relative dose intensity (RDI). Statistical analyses included least absolute shrinkage and selection operator (LASSO) regression to identify the key predictors of RDI and Cox proportional hazards models and assess the impact on survival. Results: Overall, 116 patients were included in this analysis. In the multivariate analysis using LASSO regression, higher GLIM severity was independently associated with a lower RDI (coefficient = −0.0216; *p* < 0.01). Other significant factors influencing RDI included older age (*p* < 0.01), female sex (*p* = 0.02), higher mGPS (*p* = 0.03), higher CONUT score (*p* = 0.04), and higher CAR (*p* = 0.05), all of which were associated with a lower RDI. The Cox proportional hazards analysis revealed that higher RDI was significantly associated with better survival (hazard ratio [HR] = 0.06; *p* < 0.005). Conclusions: This study highlights the critical role of immunonutritional status, particularly as measured using the GLIM criteria, in maintaining adherence to chemotherapy and improving survival outcomes in patients with gastric cancer. Routine preoperative nutritional assessments using GLIM can help identify high-risk patients, and early nutritional interventions may improve chemotherapy adherence and outcomes. These findings support the integration of nutritional strategies, specifically targeting those identified by the GLIM, into standard care to enhance the efficacy and survival of chemotherapy.

## 1. Introduction

Gastric cancer is a significant global health issue, ranking fifth in incidence and fourth in mortality, with nearly one million new cases and 800,000 deaths annually [1]. The disease burden is particularly high in East Asia, including Korea, Japan, and China. Despite advancements in diagnostic and therapeutic strategies, the prognosis for advanced-stage gastric cancer remains poor [1,2]. The 5-year survival rate for stage II gastric cancer is approximately 60–70%, and for stage III gastric cancer, it is approximately 20–40% [3].

Adjuvant chemotherapy is crucial for managing stages II and III gastric cancer and improving overall survival (OS) and disease-free survival after curative gastrectomy [4]. The XELOX regimen, comprising capecitabine and oxaliplatin, is widely accepted [5]. However, its efficacy depends on the patient’s adherence to the chemotherapy schedule, commonly referred to as chemoadherence. Poor adherence can reduce the treatment efficacy and lead to suboptimal outcomes [6,7,8]. Many patients do not complete their chemotherapy regimen owing to postsurgical complications, poor nutritional status, and the adverse effects of chemotherapy [9,10]. Reportedly, only 60–70% of patients complete their adjuvant chemotherapy regimens [11].

Disease-related malnutrition (DRM) is a form of malnutrition caused by concomitant diseases, such as cancer, and is linked to a reduction in energy–protein intake and the presence of chronic or acute inflammation [12]. Cancer patients are at high risk for malnutrition, with up to 40% affected at diagnosis, and the prevalence increasing to 80% in advanced stages [13]. In oncological patients, managing nutritional status before surgery is critical for optimizing recovery and treatment outcomes. Recent advances in oncologic nutrition have highlighted the critical role of immunonutritional status in influencing chemotherapy outcomes and survival [14]; however, a critical gap remains in our understanding of these factors in gastric cancer [9]. Postgastrectomy patients are particularly vulnerable to malnutrition, which significantly hinders recovery and chemotherapy efficacy [15,16]. Despite the established prognostic value of biomarkers, such as the C-reactive protein-to-albumin ratio (CAR), neutrophil-to-lymphocyte ratio (NLR), prognostic nutritional index (PNI), controlling nutritional status (CONUT) score, and modified Glasgow Prognostic Score (mGPS) across various malignancies [17,18,19,20], their impact on chemoadherence and survival remains unexplored in gastric cancer patients treated with the XELOX regimen.

The Global Leadership Initiative on Malnutrition (GLIM) was established in collaboration with leading international clinical nutrition societies including the European Society for Clinical Nutrition and Metabolism (ESPEN), American Society for Parenteral and Enteral Nutrition (ASPEN), and Asian Society of Parenteral and Enteral Nutrition (PENSA) [21]. A significant difference between the GLIM criteria lies in their emphasis on body composition, particularly the assessment of muscle mass, which is increasingly recognized as a critical component of nutritional status. This is particularly relevant in oncology, where muscle wasting is a common issue that can significantly affect prognosis and treatment outcomes [10]. However, research on the application of the GLIM criteria to predict chemoadherence and survival in patients with gastric cancer undergoing XELOX chemotherapy remains limited.

This study aimed to bridge this gap in the literature by evaluating the prognostic significance of the GLIM criteria alongside established immunonutritional indices in patients with stage II/III gastric cancer receiving XELOX chemotherapy. Specifically, we investigated how immunonutritional status influences chemoadherence and examined whether these factors collectively impact survival outcomes. By linking immunonutritional status to chemoadherence and, ultimately, to OS, this study aimed to provide a comprehensive understanding of the interplay among these factors, offering insights that could refine therapeutic strategies and enhance clinical outcomes in gastric cancer.

## 2. Materials and Methods

### 2.1. Population

This retrospective study analyzed patients who underwent gastrectomy for gastric cancer between January 2013 and December 2018 at the Soonchunhyang University Cheonan Hospital. Patients who underwent curative gastrectomy and were pathologically confirmed to have stage II or III disease, based on the 7th edition of the *Cancer Staging Manual* of the American Joint Committee on Cancer (AJCC), were considered for this study. We included patients who received adjuvant chemotherapy, were treated with the XELOX regimen, and completed at least one cycle of chemotherapy. Patients with a history of treatment for other malignant diseases, those who underwent surgery for gastric cancer complications, such as bleeding or obstruction, and those who had received neoadjuvant chemotherapy were excluded. The follow-up time was calculated from the date of surgery to the last known visit. In cases where patients were lost to follow-up, they were censored at the time of their last known contact for the purposes of survival analysis.

### 2.2. Chemotherapy Adherence

Chemotherapy was initiated within 6–8 weeks after surgery, and the XELOX regimen was administered as follows: capecitabine 1000 mg/m^2^ twice daily on days 1–14 and oxaliplatin 130 mg/m^2^ on day 1, repeated every three weeks for eight cycles, based on the CLASSIC trial protocol [5]. Early discontinuation was defined as receiving < four cycles of chemotherapy [22]. The relative dose intensity (RDI) was used to evaluate chemotherapy adherence [6]. RDI was calculated as the actual dose intensity/projected dose intensity for each drug separately in the treatment regimen, and the total RDI was calculated as the average of the RDI values for all medications. Dose intensity was expressed as total drug (mg)/body surface area (m^2^) × number of weeks. RDI was divided into two categories as follows: high (≥0.8) and low (<0.8) [23].

### 2.3. Body Composition Assessment

**Figure 1:**
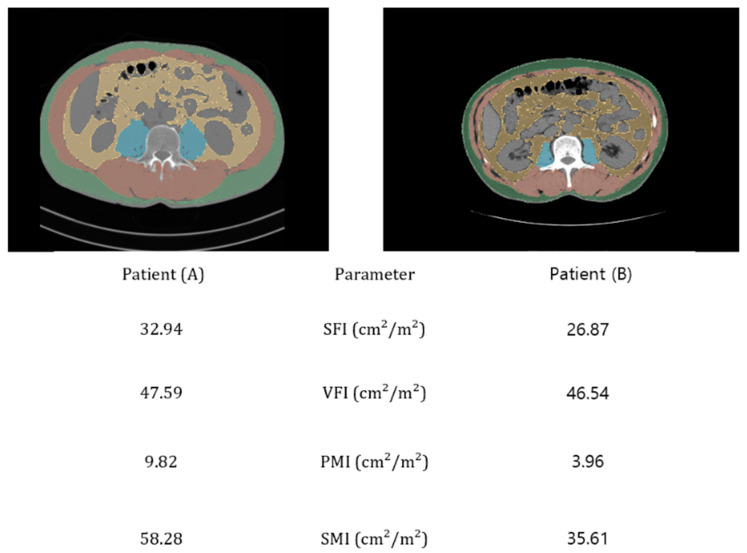
Body composition analysis at the L3 level using abdominal pelvic CT (APCT). Evaluation of the skeletal muscle index (SMI), psoas muscle index (PMI), subcutaneous fat index (SFI), and visceral fat index (VFI) areas using non-contrast-enhanced APCT imaging. The figure compares a patient with severely reduced muscle mass according to the GLIM criteria (**B**) against a patient with normal muscle mass (**A**).

Preoperative non-contrast-enhanced CT scans (GoldSeal CT750; GE Healthcare, Chicago, IL, USA) were used to evaluate body composition prior to surgery. The analysis was conducted using 3D Slicer Software (version 5.2, www.slicer.org, accessed January 2024), where semi-automated segmentation techniques were applied to measure key areas of interest, including total skeletal muscle area (SMA), psoas muscle area (PMA), subcutaneous adipose tissue (SAT), and visceral adipose tissue (VAT). These measurements were obtained from a single axial slice at the level of the L3 vertebra (Figure 1). The regions of interest (ROI) were delineated based on anatomical landmarks and standardized Hounsfield units (HUs). The total SMA included muscles such as the psoas, lumbar erector spinae, quadratus lumborum, transversus abdominis, internal and external oblique, and rectus abdominis, segmented using a CT attenuation threshold of −29 to 150 HU. The PMA was identified within the same threshold range. SAT and VAT were measured with CT-attenuation thresholds of −190 to −30 HU and −150 to −50 HU, respectively [24].

All measurements for SMA, PMA, SAT, and VAT were standardized by dividing them by the square of the patient’s height (m^2^) to produce indices such as the visceral fat index (VFI), subcutaneous fat index (SFI), skeletal muscle index (SMI), and psoas muscle index (PMI), reported in cm^2^/m^2^. These indices were used to assess the nutritional and physical status of the patients prior to surgery.

### 2.4. GLIM Criteria

The nutritional status of patients before surgery was evaluated using the GLIM criteria, which define malnutrition based on a combination of phenotypic and etiologic factors.

All clinical and laboratory data, including GLIM criteria and immunonutritional indices, were retrospectively obtained from perioperative patients’ electronic medical records. According to the GLIM criteria, malnutrition can be further classified into stage 1 (moderate) and stage 2 (severe), based on the severity of the phenotypic criterion. Patients meeting at least one phenotypic criterion indicating a mild reduction were categorized as having stage 1 (moderate) malnutrition, whereas those meeting the threshold for severe reduction were categorized as having stage 2 (severe) malnutrition. Patients meeting at least one phenotypic and etiological criterion were categorized as malnourished, whereas those who did not meet these criteria were classified as non-malnourished [25].

The phenotypic criteria included weight loss (>5% within the past 6 months or >10% beyond 6 months), low body mass index (BMI < 18.5 kg/m^2^ if <70 years or <20 kg/m^2^ if ≥70 years for Asians), and reduced muscle mass. Reduced muscle mass was further graded into mild and severe categories as follows: mild reduction was defined as a skeletal muscle index of <46.7 cm^2^/m^2^ for men and <33.6 cm^2^/m^2^ for women, while severe reduction was defined as an index of ≤39.8 cm^2^/m^2^ for men and ≤28.5 cm^2^/m^2^ for women. These criteria for reduced muscle mass were based on reference values from a study using Korean data [26].

The etiologic criteria included reduced food intake (≤50% of energy requirements for >1 week, any reduction for >2 weeks, or any gastrointestinal condition that adversely impacts food absorption) and inflammation (acute disease/injury or chronic disease-related condition such as malignant disease).

#### Other Immunonutritional Indices

The other immunonutritional indices used in this study included the following: PNI: 10 × serum albumin (g/dL) + 0.005 × total lymphocyte count (/mm^3^), NLR: neutrophil count (/mm^3^)/lymphocyte count (/mm^3^), CAR: C-reactive protein (mg/dL)/serum albumin (g/dL), mGPS: score 0: CRP ≤ 1.0 mg/dL and albumin ≥ 3.5 g/dL; score 1: CRP > 1.0 mg/dL or albumin < 3.5 g/dL; score 2: CRP > 1.0 mg/dL and albumin < 3.5 g/dL; CONUT score: albumin (g/dL): ≥3.5 (0 points), 3.0–3.49 (2 points), 2.5–2.99 (4 points), and <2.5 (6 points); total cholesterol (mg/dL): ≥180 (0 points), 140–179 (1 point), 100–139 (2 points), and <100 (3 points); and lymphocyte count (/mm^3^): ≥1600 (0 points), 1200–1599 (1 point), 800–1199 (2 points), and <800 (3 points).

### 2.5. Statistical Analysis

All analyses were performed using R version 4.1.2 (R Foundation for Statistical Computing, Vienna, Austria). Descriptive statistics were used for categorical and continuous variables. Normality was assessed using the Shapiro–Wilk test, and appropriate parametric or non-parametric tests were applied based on the results. To address multicollinearity, least absolute shrinkage and selection operator (LASSO) regression was employed for variable selection. For survival analysis, both the Kaplan–Meier method and Cox proportional hazards model were utilized. Graphical representations were created using the ggplot2 package.

## 3. Results

**Figure 2:**
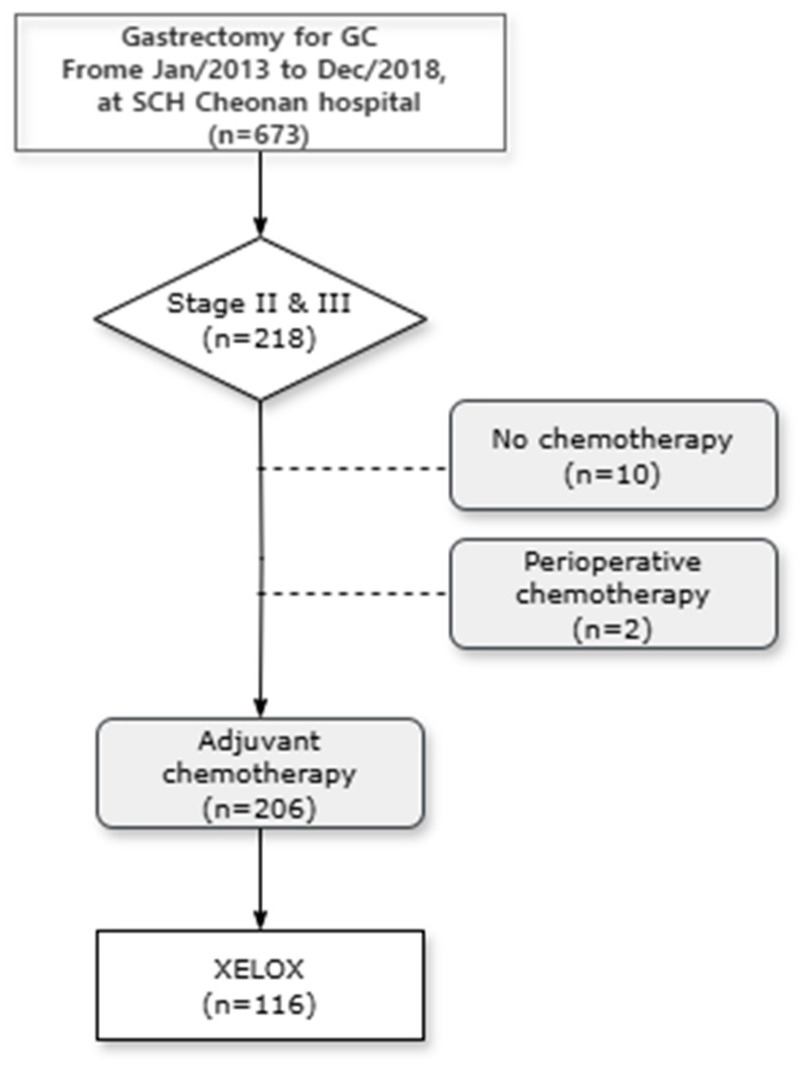
Study flowchart of patients with gastric cancer who underwent gastrectomy.

Overall, 673 patients were included in this study, 218 of whom were diagnosed with stage II or III gastric cancer. From this group, we excluded ten patients who did not receive adjuvant chemotherapy and two who underwent perioperative chemotherapy. After excluding patients who received adjuvant chemotherapy with regimens other than XELOX, 116 patients were included in the final analysis (Figure 2).

The baseline clinical characteristics of the 116 patients included in this study are summarized in Table 1. The mean age of the cohort was 59.35 ± 10.98 years, and the mean BMI was 23.57 ± 3.24 kg/m^2^. The sex distribution consisted of 88 men (75.86%) and 28 women (24.14%). Regarding the extent of resection, 84 (72.41%), 30 (25.86%), and 2 (1.72%) patients underwent distal, total, and proximal gastrectomy. The pathological staging of gastric cancer was as follows: 64 patients (55.17%) were classified as stage II and 52 patients (44.83%) were classified as stage III. Postoperative complications within 30 days occurred in one patient (0.86%) who developed pneumonia. The mean time to initiation of adjuvant chemotherapy (TTAC) was 38.41 ± 8.63 days, and the mean weight at initiation of chemotherapy (WIC) was 58.44 ± 10.24 kg. In analyzing chemotherapy-related metrics, 32 (27.6%) of the 116 patients completed treatment without any dose reduction or interruption. The remaining 84 patients (72.4%) required at least one intervention (dose reduction or treatment interruption) for the reasons detailed in Table 2. Based on an RDI threshold of 0.8, 69 patients (59.5%) were categorized as having a high RDI. The median follow-up time was 61.00 months (range: 2.00–113.00 months). Patients who were lost to follow-up were censored at their last known contact.

The following section provides a summary of the descriptive statistics related to nutritional indicators and body composition (Table 3). Based on the GLIM criteria, patients were categorized into no malnutrition (n = 51), moderate malnutrition (n = 43), and severe malnutrition (n = 22) groups with corresponding mean RDIs of 0.84, 0.77, and 0.70, respectively. For PNI, patients were grouped into PNI ≥ 45 (n = 96), with a mean RDI of 0.811 ± 0.182, and PNI < 45 (n = 20), with a mean RDI of 0.687 ± 0.268, using the established cutoff value of 45 [27]. Patients were categorized by the CONUT score into low-risk (0–1, n = 63) and high-risk (≥2, n = 53) groups, with mean RDIs of 0.806 ± 0.201 and 0.778 ± 0.199, respectively. The mGPS groups showed a mean RDI of 0.814 ± 0.188 for mGPS 0 (n = 95), 0.710 ± 0.218 for mGPS 1 (n = 17), and 0.555 ± 0.314 for mGPS 2 (n = 4). The mean CAR was 0.141 ± 0.238. When adjusted for body surface area, the VFI and SFI were 39.18 ± 25.86 cm^2^/m^2^ and 43.72 ± 22.26 cm^2^/m^2^, respectively. Additionally, the PMI was 5.70 ± 1.70 cm^2^/m^2^, and the SMI was determined to be 46.54 ± 9.01 cm^2^/m^2^.

**Figure 3:**
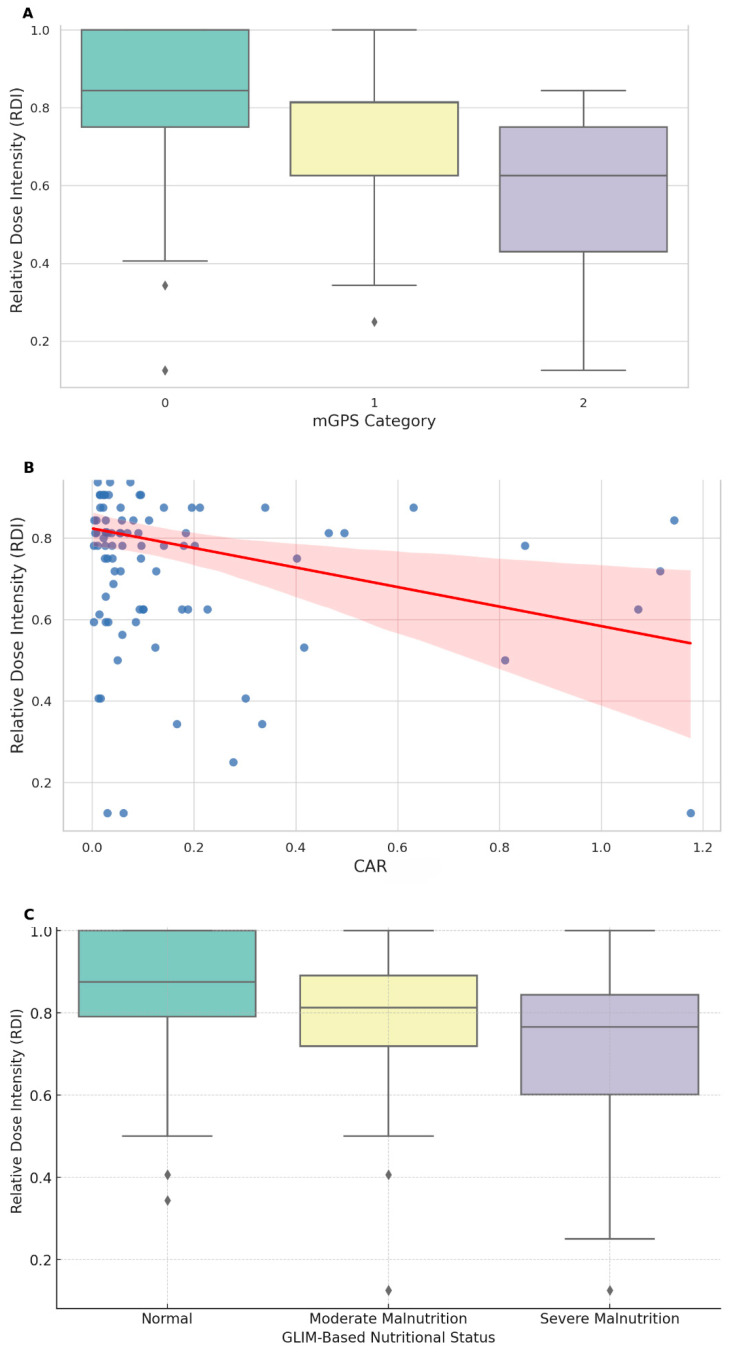
Univariate analysis of the associations between nutritional inflammatory indices and relative dose intensity (RDI). (**A**)**:** The modified Glasgow Prognostic Score (mGPS) was significantly negatively associated with RDI (H = 7.473, *p* = 0.024). (**B**)**:** The C-reactive protein-to-albumin ratio (CAR) showed a significant negative correlation with RDI (Spearman’s r = −0.285, *p* = 0.0019). (**C**)**:** The Global Leadership Initiative on Malnutrition (GLIM) criteria showed a significant decrease in RDI with increasing malnutrition severity (H = 9.468, *p* = 0.0088).

In the univariate analysis, age was significantly negatively correlated with RDI (Spearman’s r = −0.281, *p* = 0.0029), indicating a lower RDI in older patients. Stage was not significantly associated with RDI (Mann–Whitney U = 1602.0, *p* = 0.7306), and BMI showed a weak, nonsignificant positive correlation with RDI (Spearman’s r = 0.177, *p* = 0.065). Among the biochemical markers, TIBC was positively correlated with RDI (Spearman’s r = 0.209, *p* = 0.0288), whereas CRP and hemoglobin levels were significantly negatively correlated (Spearman’s r = −0.279 and −0.217, *p* = 0.002 and 0.019, respectively). mGPS was significantly negatively associated with RDI (H = 7.473, *p* = 0.024) (Figure 3A), and CAR was significantly negatively correlated with RDI (Spearman’s r = −0.285, *p* = 0.0019) (Figure 3B). The GLIM criteria showed a significant decrease in RDI with increasing malnutrition severity (H = 9.468, *p* = 0.0088) (Figure 3C) and no significant difference in RDI between the low-risk (0–1) and high-risk (≥2) CONUT groups (*p* = 0.240). However, PNI showed a nonsignificant trend toward a lower RDI in patients with scores <45 (*p* = 0.0627), and no significant correlation was found with the NLR. The body composition analysis revealed a significant positive correlation between SMI and RDI (r = 0.350, *p* < 0.001) and a weaker but significant correlation between PMI and RDI (r = 0.263, *p* = 0.005), whereas VFI and SFI did not significantly correlate with RDI.

**Figure 4:**
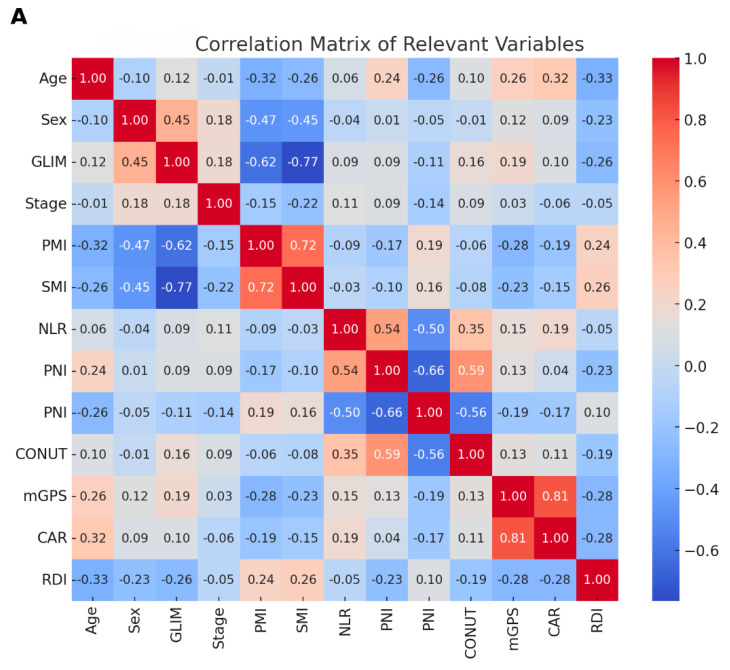

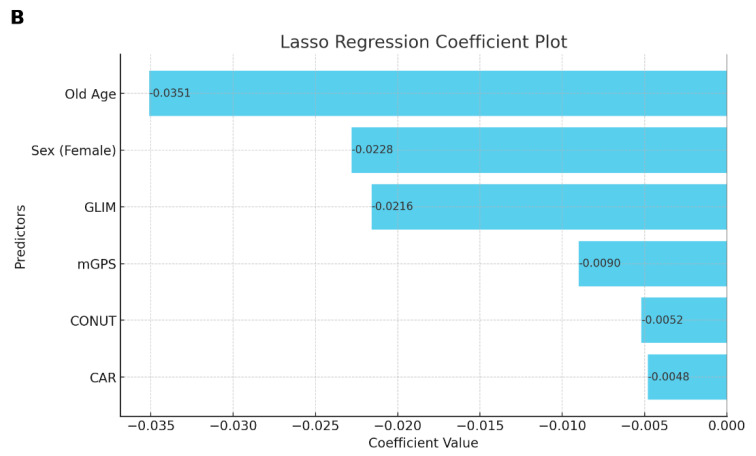
Correlation matrix and LASSO regression analysis of predictors related to RDI. (**A**) Correlation matrix of relevant clinical and nutritional variables, including age, sex, GLIM criteria, stage, psoas muscle index (PMI), skeletal muscle index (SMI), neutrophil-to-lymphocyte ratio (NLR), prognostic nutritional index (PNI), controlling nutritional status (CONUT), modified Glasgow Prognostic Score (mGPS), C-reactive protein-to-albumin ratio (CAR), and relative dose intensity (RDI). The color gradient represents the strength and direction of the correlation (from −1.0 to +1.0), (**B**) LASSO (least absolute shrinkage and selection operator) regression coefficient plot showing the influence of significant predictors on RDI. The variables include age, sex (female), GLIM criteria, mGPS, CONUT, and CAR, with negative coefficients indicating a stronger association with lower RDI.

We employed LASSO regression for multivariate analysis to identify and select the key factors influencing RDI while minimizing overfitting. Variables that were significant in the univariate analysis, along with key clinical factors such as age, sex, and stage, were included in the model. Age emerged as a significant predictor, with older patients showing lower RDI values (coefficient = −0.0351). Female sex was also associated with a reduction in RDI compared with male sex (coefficient = −0.0228). Additionally, the GLIM criteria for malnutrition significantly affected RDI, with higher GLIM scores linked to a lower RDI (coefficient = −0.0216). Other nutritional and inflammatory markers, such as mGPS (coefficient = −0.0090), CONUT (coefficient = −0.0052), and CAR (coefficient = −0.0048), were also negatively associated with RDI. A correlation matrix analysis further supported these findings, revealing generally low-to-moderate correlations among the variables, suggesting that multicollinearity was unlikely to impact the regression results significantly (Figure 4A). While the LASSO model’s overall explanatory power was modest, the identified predictors were clinically relevant and should be considered when optimizing treatment strategies to improve patient outcomes (Figure 4B).

In the Cox proportional hazards model, both RDI and disease stage were significant independent predictors of survival. Specifically, a higher RDI was associated with significantly better survival (HR = 0.06, 95% CI: 0.01–0.40, *p* < 0.005). Conversely, advancing disease stage was associated with significantly worse survival (HR = 4.13, 95% CI: 1.57–10.88, *p* < 0.005). The model demonstrated good predictive accuracy with a concordance index of 0.72, and the overall model fit was statistically significant (log-likelihood ratio test, *p* < 0.005).

**Figure 5:**
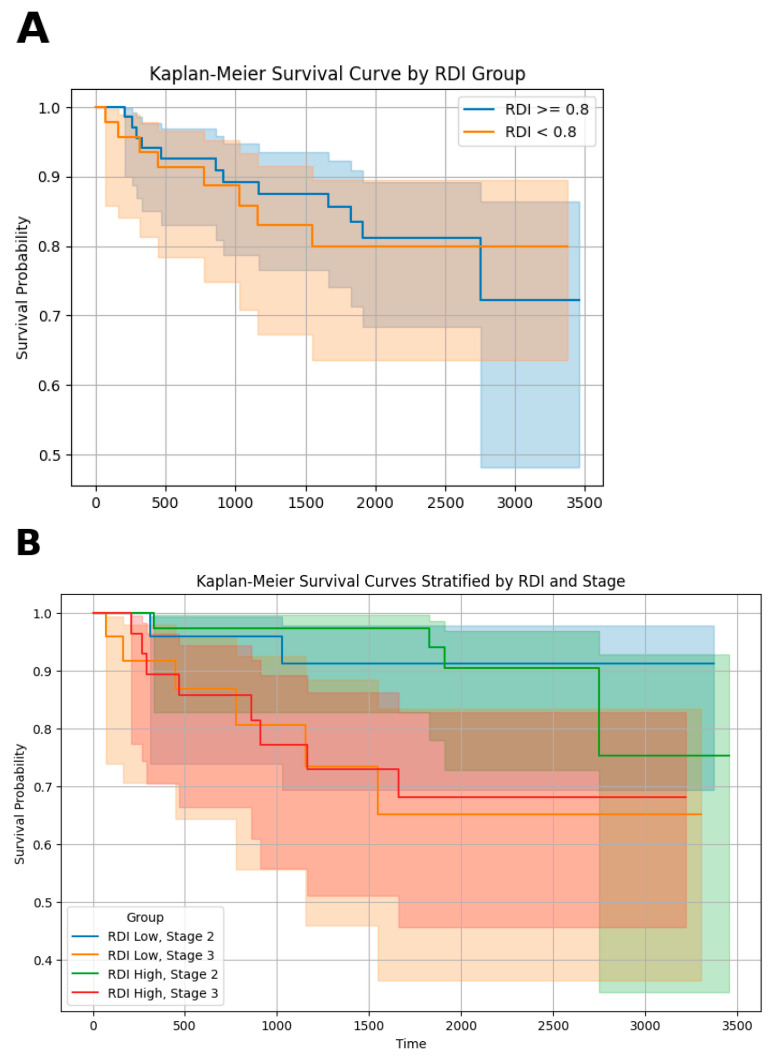
Kaplan–Meier survival curves based on RDI and stage. (**A**) Kaplan–Meier survival curve showing the difference in survival probability between patients with relative dose intensity (RDI) ≥ 0.8 and those with RDI < 0.8. Shaded areas represent the 95% confidence intervals for each group, (**B**) Kaplan–Meier survival curves stratified by both RDI (high vs. low) and cancer stage (stage II vs. stage III). The survival curves compare the following four groups: RDI low, stage II; RDI low, stage III; RDI high, stage II; and RDI high, stage III. Shaded areas represent the 95% confidence intervals for each group.

Kaplan–Meier survival curves stratified by RDI and disease stage demonstrated clear differences in survival probabilities across the groups. Patients with a higher RDI (>0.8) consistently showed better survival outcomes compared with those with a lower RDI (≤0.8) (Figure 5A). Specifically, the RDI high and stage II groups exhibited the highest survival probability over time, whereas the RDI low and stage III groups had the lowest survival probabilities (Figure 5B).

## 4. Discussion

The high prevalence of DRM in cancer patients highlights the importance of addressing both nutritional intake and inflammation [13]. In cancer patients, particularly those undergoing major surgeries such as gastrectomy, immunonutrition has the potential to modulate the inflammatory response and improve patient recovery [28]. The relationship between treatment outcomes and immunonutritional status, including body composition, has gained significant attention in oncology, owing to its profound impact on treatment outcomes [29]. However, specific research examining these relationships in gastric cancer remains limited, particularly within East Asian populations where the disease is most prevalent. This gap is concerning, given the severe nutritional deterioration commonly associated with advanced-stage gastric cancer, which can further exacerbate the already low survival rates in these patients.

This study aimed to fill this gap by investigating the relationships among nutritional status, RDI, and survival outcomes in patients undergoing XELOX chemotherapy. XELOX refers to a combination chemotherapy regimen that includes capecitabine (Xeloda) and oxaliplatin, commonly used in the treatment of advanced gastric cancer [5]. XELOX works by inhibiting cancer cell proliferation, but its cytotoxic effects can also impact the patient’s nutritional status, leading to side effects such as nausea, vomiting, and appetite loss. These factors are important when analyzing correlations with the patient’s nutritional status, as chemotherapy-induced malnutrition can further affect treatment outcomes. Building on previous research [30] in which we demonstrated the impact of body composition and immunonutrition on chemoadherence in colorectal cancer, we chose to focus on the GLIM criteria. These criteria include body composition as a key component in assessing nutritional status, offering a comprehensive and validated approach for evaluating the impact of nutrition on RDI and survival.

In this study, we identified several clinical and nutritional factors that significantly influenced RDI during chemotherapy. Our findings underscore the critical roles of patient demographics and nutritional status in maintaining patient adherence to chemotherapy. Notably, age and albumin, CRP, and hemoglobin levels were inversely correlated with RDI, which aligns with existing evidence suggesting that older patients and those with poorer nutritional status are at a greater risk of reduced chemotherapy dosing. The positive association between TIBC and RDI suggests that better iron status may contribute to improved chemotherapy adherence. Previous research has highlighted the importance of iron status in gastric cancer [31], which may significantly influence chemotherapy adherence in post-surgical patients. However, further studies are required to elucidate these mechanisms.

Through univariate analysis, we observed that muscle mass significantly affected the RDI in gastric cancer, which is consistent with our previous findings in patients with colorectal cancer [30]. Both the PMI and SMI were statistically significant, supporting the clinical utility of the GLIM as an index for assessing malnutrition in predicting chemoadherence.

Moreover, the multivariate analysis revealed that several factors independently contributed to the variation in RDI, highlighting the complex interplay between demographic, nutritional, and inflammatory factors in chemotherapy tolerance. Age and female sex were negatively associated with RDI, suggesting that older patients and women are at a higher risk of reduced dose intensity, likely because of age-related physiological decline and sex-specific differences in chemotherapy metabolism and toxicity [22,32,33]. Furthermore, the significant impact of nutritional and inflammatory markers, such as GLIM, mGPS, CONUT, and CAR, on RDI emphasizes the importance of addressing malnutrition and systemic inflammation to improve treatment adherence [34]. These findings underscore the need for a personalized approach to chemotherapy that integrates comprehensive nutritional and inflammatory assessments, particularly for vulnerable populations, such as malnourished elderly women, to optimize dose intensity and enhance overall treatment outcomes. While the role of immunonutrition in enhancing chemotherapy adherence and survival requires further exploration, its integration into standard care could lead to improved outcomes for patients undergoing chemotherapy.

A key strength of our study is the use of Korea-specific data for the assessment of sarcopenia [26], which distinguishes our study from other studies that rely on generalized or non-population-specific standards. This approach not only enhances the accuracy of our findings but also aligns with the strengths of the GLIM criteria, which emphasize a multidimensional assessment of nutritional status that includes body composition. By integrating population-specific data with a comprehensive GLIM framework, our study provides a more precise evaluation of sarcopenia and its implications in the Korean population, thereby contributing valuable insights to the field.

Although a high RDI is widely recognized as beneficial in various cancers, research on gastric cancer, particularly in the adjuvant setting with XELOX, is limited [35,36,37]. Our analysis indicated a significant relationship between the RDI and survival outcomes in patients receiving XELOX chemotherapy for advanced gastric cancer. Patients in stage II who maintain an RDI above 0.8 experience the best survival outcomes, while those in stage III with a lower RDI face the greatest risk. These findings contribute to the limited research on RDI’s impact on gastric cancer and highlight the importance of strategies aimed at sustaining high-dose intensity during treatment, particularly for those with advanced disease stages.

While previous research has demonstrated a direct link between nutritional markers, such as PNI, CAR, CONUT score, and mGPS, and survival in patients [38,39,40,41], our findings suggest a more nuanced relationship. Specifically, although we confirmed an association between poor nutritional status and low RDI, we did not observe a statistically significant direct link between these nutritional markers and OS. This finding contrasts with those of many previous studies and highlights the complexity of the relationship between nutrition and survival outcomes.

Our previous study demonstrated that the GLIM criteria significantly impact recurrence-free survival (RFS) in gastric cancer [42]. However, in the present study, we found that the GLIM criteria did not have a statistically significant effect on OS. This discrepancy may be attributed to several key differences between the studies. First, the previous study included patients with stage I disease, whereas the current study focused exclusively on patients with advanced gastric cancer. Early-stage patients with a lower risk of recurrence may have been more susceptible to changes in nutritional status, which could have had a greater impact on RFS. In contrast, in advanced-stage patients, factors such as tumor biology and treatment response may have played a more dominant role in influencing survival. Additionally, a previous study used RFS as the primary endpoint, which may have better reflected the influence of nutritional status and body composition on postsurgical recovery and tumor regrowth. In contrast, OS includes all-cause mortality, which potentially dilutes the impact of the GLIM criteria. These findings suggest that while the GLIM criteria may be useful for assessing recurrence risk, they may have limitations as standalone predictors of OS in patients with advanced gastric cancer.

Despite these significant findings, this study has some limitations. As a retrospective study, it is subject to biases in data collection and patient selection. Notably, variables such as psychological distress during treatment and household income, which could influence chemotherapy adherence, were not systematically collected. Although most patients were covered by the National Health Insurance (NHI), reducing disparities in access to chemotherapy, income-related treatment interruptions cannot be entirely ruled out. Being conducted at a single center may also limit the generalizability of the results. Additionally, unmeasured confounding variables may have influenced the observed associations, and the relatively small sample size suggests that larger prospective studies are required to confirm our findings. This study provides a comprehensive understanding of how nutritional status affects survival.

## 5. Conclusions

In conclusion, our study provides compelling evidence that nutritional status and body composition are critical determinants of chemotherapy adherence in patients with advanced gastric cancer. By maintaining a higher RDI through the optimization of nutritional and muscle health, survival outcomes in this patient population can be significantly improved. Clinically, this emphasizes the need for routine preoperative assessments of nutritional status and body composition using tools like the GLIM criteria. Early identification of malnutrition and targeted nutritional support should be integrated into standard care protocols to enhance chemotherapy adherence. These findings support the integration of nutritional strategies, including the use of body composition-reflective indicators such as GLIM, into the standard care of patients with gastric cancer. Further research is warranted to explore the potential benefits of targeted nutritional and exercise interventions in enhancing chemotherapy efficacy and patient survival.

## Figures and Tables

**Table 1:** Baseline characteristics.

Variable	No Malnutrition	Moderate	Severe	Total
	(N = 51)	(N = 43)	(N = 22)	(N = 116)
**Age**	58.43 ± 8.39	58.98 ± 11.85	62.23 ± 14.17	59.35 ± 10.98
**BMI**	25.21 ± 2.96	23.12 ± 2.57	20.67 ± 2.75	23.57 ± 3.24
**Sex**				
Female	3 (5.89%)	12 (27.91%)	13 (59.09%)	28 (24.14%)
Male	48 (94.11%)	31 (72.09%)	9 (40.91%)	88 (75.86%)
**TTAC** **(days)**	37.61 ± 9.78	39.05 ± 6.07	39.05 ± 10.16	38.41 ± 8.63
**WIC** **(kg)**	63.90 ± 10.33	56.49 ± 6.84	49.59 ± 8.04	58.44 ± 10.24
**Number of retrived lymph nodes**				39.58 ± 13.06
**Surgical approach**				
Open	48 (90.56%)	39 (90.69%)	20 (90.90%)	105 (90.52%)
Laparoscopy	5 (9.54%)	4 (9.31%)	2 (9.10%)	11 (9.48%)
**Extent of resection**				
Distal gastrectomy	38 (74.51%)	28 (65.21%)	18 (81.82%)	84 (72.41%)
Total gastrectomy	13 (25.49%)	13 (30.23%)	4 (18.18%)	30 (25.86%)
Proximal gastrectomy		2(4.65%)		2 (1.72%)
**T stage**				
T1	5 (9.8%)	3 (6.98%)	1 (4.55%)	9 (7.76%)
T2	10 (19.61%)	5 (11.63%)	4 (18.18%)	19 (16.38%)
T3	23 (45.1%)	22 (51.16%)	10 (45.45%)	55 (47.41%)
T4	13 (25.49%)	13 (30.24%)	7 (31.82%)	33 (28.45%)
**N_stage**				
N0	11 (21.57%)	10 (23.26%)	3 (13.64%)	24 (20.69%)
N1	13 (25.49%)	13 (30.23%)	6 (27.27%)	32 (27.59%)
N2	12 (23.53%)	9 (20.93%)	4 (18.18%)	25 (21.55%)
N3	15 (29.41%)	11 (25.58%)	9 (40.90%)	35 (30.17%)
**AJCC Stage**				
II	34 (64.15%)	24 (55.81%)	8 (36.37%)	64 (55.17%)
III	19 (35.85%)	19 (44.19%)	14 (63.63%)	52 (44.83%)
**Lymphatic invasion**				
Not identified	12 (23.53%)	16 (37.21%)	5 (22.73%)	33 (28.45%)
present	39 (76.47%)	27 (62.79%)	17 (77.27%)	83 (71.55%)
**Venous invasion**				
Not identified	42 (82.35%)	35 (81.40%)	18 (81.82%)	95 (81.90%)
present	9 (17.65%)	8 (18.60%)	4 (18.18%)	21 (18.10%)
**Perineural invasion**				
Not identified	23 (45.10%)	18 (41.86%)	11 (50.00%)	52 (44.83%)
present	28 (54.90%)	25 (58.14%)	11 (50.00%)	64 (55.17%)
**30-day postoperative complications**				
No	50	43	22	115 (99.14%)
Yes	1	0	0	1 (0.86%)

Note: No malnutrition, moderate, and severe categories are classified according to the GLIM (Global Leadership Initiative on Malnutrition) criteria. Abbreviations: TTAC, time to initiation of adjuvant chemotherapy; WIC, weight at initiation of chemotherapy.

**Table 2:** Reasons for treatment modifications and interventions.

Reason for Intervention (CTCAE Version 5.0 Terminology)	No Malnutrion (N = 30)	Moderate (N = 33)	Severe (N = 21)	Total (N = 84)
Blood and lymphatic system disorders	1	12	8	21
Nausea and vomiting	15	6	3	24
Peripheral sensory neuropathy	4	4	2	10
Diarrhea	2	3	1	6
Palmar–plantar erythrodysesthesia syndrome	1	2	0	3
Hepatobiliary disorders	0	0	1	1
Acute kidney injury	1	0	0	1
General physical health deterioration	6	6	6	18

Note: No malnutrition, moderate, and severe categories are classified according to the GLIM (Global Leadership Initiative on Malnutrition) criteria.

**Table 3:** Nutritional indicators and body composition.

Variable	No Malnutrition	Moderate	Severe	Total or Mean	RDI
	(N = 51)	(N = 43)	(N = 22)	(N = 116)	
SFA (cm^2^)	121.33 ± 59.84	115.57 ± 59.92	104.98 ± 46.81	116.10 ± 57.48	
VFA (cm^2^)	136.38 ± 79.83	91.44 ± 56.05	63.17 ± 49.66	105.84 ± 72.17	
PMA (cm^2^)	18.52 ± 4.58	14.14 ± 3.30	10.60 ± 4.66	15.40 ± 5.14	
SMA (cm^2^)	126.81 ± 19.23	105.01 ± 15.48	80.45 ± 17.99	125.33 ± 28.51	
VFI (cm^2^/m^2^)	49.38 ± 27.97	34.36 ± 21.07	24.94 ± 19.95	39.18 ± 25.86	
SFI (cm^2^/m^2^)	44.24 ± 20.50	44.26 ± 25.67	41.46 ± 19.68	43.72 ± 22.26	
PMI (cm^2^/m^2^)	6.79 ± 1.46	5.24 ± 1.06	4.07 ± 1.55	5.70 ± 1.70	
SMI (cm^2^/m^2^)	53.39 ± 6.25	44.23 ± 5.15	35.17 ± 6.17	46.54 ± 9.01	
PNI					
≥45	44 (86.27%)	35 (81.40%)	17 (77.27%)	96 (82.75%)	0.811 ± 0.182
<45	7 (13.73%)	35 (81.40%)	5 (22.73%)	20 (17.25%)	0.687 ± 0.268
NLR	1.77 ± 0.87	2.05 ± 0.91	1.90 ± 0.77	1.90 ± 0.87	
CAR	0.10 ± 0.06	0.19 ± 0.10	0.14 ± 0.09	0.14 ± 0.24	
CONUT score					
Normal (0–1)	29 (56.86%)	25 (58.14%)	9 (40.91%)	63 (54.32%)	0.806 ± 0.201
High (≥2)	22 (43.14%)	18 (41.86%)	13 (59.09%)	53 (45.68%)	0.778 ± 0.199
mGPS					
Low risk	46 (90.2%)	33 (76.74%)	16 (72.73%)	95 (81.90%)	0.814 ± 0.188
Intermediate risk	5 (9.8%)	7 (16.28%)	5 (22.73%)	17 (14.66%)	0.710 ± 0.218
High risk		3 (6.98%)	1 (4.55%)	4 (3.45%)	0.555 ± 0.314
GLIM criteria					
No malnutrition				51 (43.97%)	0.844 ± 0.173
Moderate malnutrition				43 (37.07%)	0.771 ± 0.208
Severe malnutrition				22 (18.97%)	0.702 ± 0.232
History of weight loss					
Normal	51 (100%)	30 (69.76%)	18 (81.81%)	99 (85.34%)	
Moderate	0	13 (30.24%)	3 (13.63%)	16 (13.79%)	
Severe	0	0	1(4.55%)	1 (0.86%)	
Low BMI					
Normal	51 (100%)	41 (95.34%)	14 (63.63%)	106 (91.38%)	
Moderate	0	2 (4.66%)	6 (27.27%)	8 (6.90%)	
Severe	0	0	2 (9.09%)	2 (1.72%)	
Reduced muscle mass					
Normal	51 (100%)	8 (18.61%)	1 (4.55%)	60 (51.72%)	
Moderate	0	35 (81.39%)	0	35 (30.17%)	
Severe	0	0	21 (95.45%)	21 (18.10%)	

Note: No malnutrition, moderate, and severe categories are classified according to the GLIM (Global Leadership Initiative on Malnutrition) criteria.

## Data Availability

The datasets generated during and/or analyzed during the current study are available from the corresponding authors upon reasonable request.
